# Influence of electrochemical co-deposition of bimetallic Pt–Pd nanoclusters on polypyrrole modified ITO for enhanced oxidation of 4-(hydroxymethyl) pyridine

**DOI:** 10.1039/d2ra02510h

**Published:** 2022-06-08

**Authors:** Bharath M, Agnus T. Mathew, Akshaya K B, Uraiwan Sirimahachai, Anitha Varghese, Gurumurthy Hegde

**Affiliations:** Department of Chemistry, CHRIST (Deemed to be University) Hosur Road Bengaluru 560029 India anitha.varghese@christuniversity.in murthyhegde@gmail.com; Center of Excellence for Innovation in Chemistry and Division of Physical Science, Faculty of Science, Prince of Songkla University Hat Yai Songkhla 90110 Thailand; Centre for Advanced Research and Development (CARD), CHRIST (Deemed to be University) Hosur Road Bengaluru 560029 India

## Abstract

Bimetallic Pt–Pd nanoparticles were dispersed on polypyrrole coated indium-tin oxide coated polyethylene terephthalate sheets (ITO-PET sheets). The excellent filming property of pyrrole gives a high porous uniform active area for the proper adsorption of bimetallic transition metal nanoparticles. Electrochemical behavior of the modified electrodes was determined using cyclic voltammetry and impedance studies. The physicochemical properties of the modified electrodes were analyzed by scanning electron microscopy, X-ray diffraction spectroscopy, X-ray photoelectron spectroscopy and Fourier transform infrared spectroscopy. To study the electrochemical oxidation of 4-(hydroxymethyl) pyridine in the presence of sodium nitrate in aqueous acidic medium, the modified electrode was used. It is evident from the study that the modified electrode shows better electrochemical activity towards the oxidation of 4-(hydroxymethyl) pyridine.

## Abstract 2

Bimetallic Pt–Pd nanoparticles were dispersed on polypyrrole coated indium–tin oxide coated polyethylene terephthalate sheets (ITO-PET sheets).

## Graphical abstract

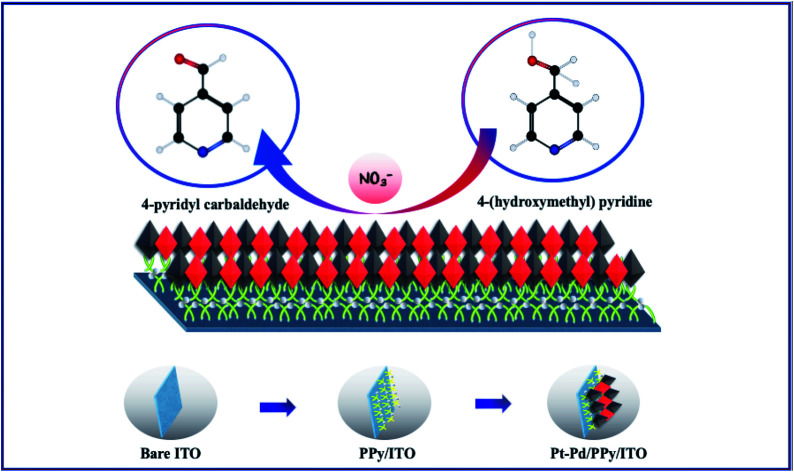

## 1 Introduction

The last few decades have seen significant research on oxidation of heterocyclic alcohols, with numerous studies undertaken in their chemical conversion to various biologically important compounds.^1^ Considering their potential applications, an access to a class of functionalized heterocyclic compounds is crucial though they have a vital role in the discovery and development of drugs.^2,3^ Rather than concentrating in synthesis and functionalization of heterocycles, there remains a great need for further advancements in their interconversion. Chemical oxidation and reduction reactions are promising methods for the synthesis of a huge variety of compounds. At the same time many among them are tedious and make use of chemically potent reagents. The electrochemical approach using an electrocatalyst replaces a chemical oxidant or reductant from the reaction mixture and ensures a clean reaction setup.

The present work emphasizes on the electrochemical oxidation of 4-(hydroxymethyl) pyridine to 4-formyl pyridine using novel modified electrode. It is evident from recent studies that conducting polymers show excellent catalytic property towards the electrocatalytic oxidation. Intrinsically conducting polymers are the materials of good conductivity and act as a supporting material as a catalyst such as polyacetylene (PA), polypyrrole (PPy), polyaniline (PANI), poly(3,4-ethylenedioxythiophene) (PEDOT), and polythiophene (PTh).^4–6^ Electrochemical deposition of conducting polymers resulted in an increase in electro-active surface area, due to increase in porosity and roughness.^7^ Polypyrrole due to its wide variety of usage such as corrosion protective coating, electrochemical sensing, and energy storage devices. Synthesis of such materials are quite possible in aqueous based medium and are quite stable enough at ambient conditions.^8–10^ Presence of conductive ions in reaction medium even act as initiator for the polymerization of monomer though it proceeds through cationic polymerization. An ion which has relatively high oxidation potential than the monomer unit will result in the oxidation of the monomer. Radical cation thus formed combines with similar units, resulting in the creation of a polymer chain.^9,10^ The presence of an acid can provide conductive ions and experimental data proves that the polymerization of pyrrole is favorable under pH 6. *In situ* doping can also takes place if the polymerization is carried out under acid medium. Doped polymers show better catalytic activity than simple polymers. Therefore, the polymerization is carried out under sulphuric acid medium. The oxidation potential of the sulphate anion exceeds that of the pyrrole monomer, this facilitates the easy oxidation of pyrrole to form polypyrrole.

Transition metal nanoparticles on electro active support materials form the basis for solid electrocatalysts which have multiple applications including fuel production,^11^ hydrogen production,^12^ sensors,^13^ supercapacitors^14^ and as electrocatalysts.^15–17^ Dispersion of metal nanoparticles on intrinsically conducting polymers offers high electrical conductivity and good adhesive property. Combining the exceptional catalytic property of metal nanoparticles and appreciable film forming nature of the conjugated polymer open up the possibility of synthesizing promising materials for advanced electrochemical studies.^18,19^ The polymer backbone facilitates the transfer of electrons between the electrode substrate and metal nanoparticles dispersed during a redox reaction. This in turn favors interfacial properties for an electrochemical system for various reactions.^20^ This improves the electrocatalytic efficiency of the electrode and thereby efficient electro oxidation and electro reduction reactions can be carried out using these modified electrodes.

Nano porous metal nano structures have garnered significant scientific interest for their potential in practical applications, and among them nano porous platinum holds the upper hand due to its high porosity and enhanced surface area per unit volume.^21^ Various Pt based bimetallic nanoparticles have been observed to be cost effective and successful replacements for Pt catalysts.^22–25^ At the same time, introducing a secondary metal can help modify the crystallographic and electronic structure of Pt nanoparticles. Recent investigations revealed that the catalytic activity of Pt-based material is highly morphology dependent.^26^ Exposed facets on the surface of Pt nanostructures have a crucial role in ascertaining the active sites and activity. Among the several Pt based metallic nanoparticles (Pt–Pd, Pt–Cu, Pt–Ni, Pt–Co *etc.*), palladium exhibits the exact face centred cubic (fcc) cubic structure as well as a comparable, matching crystal lattice with Pt.^21^ Excellent catalytic activity of Pd makes it a better candidate for alloying with Pt.^27,28^ Aqueous medium is used to carry out the electrochemical reactions. Even though the available potential range of water is relatively narrow compared to other non-polar solvents, it has some major advantages such as it permits the use of wide variety of ions for doping, non-toxic and readily available.

**Fig. 1:**
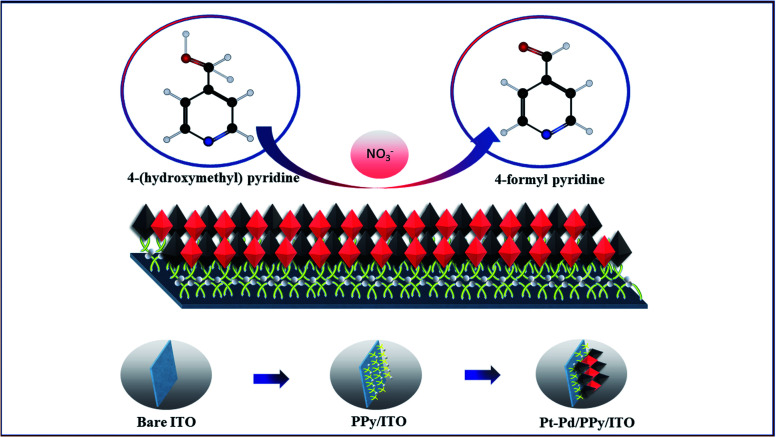
Schematic illustration of electrocatalytic oxidation of 4-(hydroxymethyl) pyridine.

In the present study, Pt–Pd nanoparticles deposited on PPy film coated ITO electrodes (Pt–Pd/PPy/ITO) have shown remarkable electrocatalytic properties towards the oxidation of 4-(hydroxymethyl) pyridine in presence of sodium nitrate as a mediator^29^ (Fig. 1). PPy-coated ITO electrode modified with electrodeposited bimetallic Pt–Pd nanoparticles are being investigated for electrocatalytic oxidation of 4-(hydroxymethyl) pyridine to 4-formyl pyridine is the first time to the best of our knowledge.

## 2. Experimental methods

### 2.1. Chemicals and reagents

An electrode is fabricated with bimetallic Pt–Pd nanoparticles dispersed over polypyrrole coated ITO electrodes. For this, analytical grade H_2_SO_4_, H_2_PtCl_6_, and PdCl_2_. 4-(Hydroxymethyl) pyridine were acquired from Sigma-Aldrich-Merck, while pyrrole monomer and sodium dodecyl sulphate (SDS, C_12_H_25_NaOSO_3_) were bought from SD Fine Chemicals Limited, India. For the experiment's measurement, a three-electrode system was employed, with modified ITO electrodes (Pt–Pd/PPy/ITO) introduced as the working electrode, platinum foil serving as the counter electrode, and calomel electrode deployed as the reference electrode. The protective covering of the ITO sheet is removed after sonicating the segmented electrode of dimension 2 cm length and 1 cm width with an additional extension. The extension was separated from the active surface with Teflon tape. All the electrochemical measurements were carried out after purging the solution with N_2_ in an air-conditioned room (22 ± 1 °C).

### 2.2 Apparatus and software

Scanning electron microscopy (SEM) images were captured using Zeiss Gemini model Ultra 55. Thermo Nicolet, Avatar 370 is used to measure Fourier Transform-Infrared (FTIR) spectra. X-Ray diffractometer (XRD) Bruker AXS D8 Advance is used to measure structural characterization of the samples having Cu Kα radiation (*λ* = 1.5406). X-ray photoelectron spectrometer (XPS) AXIS ULTRADLD, Kratos analytical, Manchester UK is used to measure the chemical composition of sample surface. The base pressure in the XPS analysis chamber will be about 5 × 10^−9^ torr. The samples are excited with X-ray hybrid mode 700 × 300 μm spot area with a monochromatic Al Kα 1, 2 radiation at 1.4 keV. X-ray anode will run at 15 kV 10 mA 150 W. The photoelectrons are detected with a hemispherical analyser positioned at an angle of 90° with respect to the normal to the sample surface. The spectra are calibrated using the C 1s line (BE = 285 eV). XPS Peak 4.1 software was used to fit the obtained results after appropriate background subtraction and Tougaard procedure with Gaussian–Lorentzian curves (70 : 30) was employed. Optimum FWHM of the peaks of different elements were maintained within optimum percentages of 1.2 eV. All the electrochemical studies were carried out using electrochemical analyser model CH1608E (CH Instruments, Inc. USA).

## 3. Results and discussion

### 3.1. Electrochemical preparation of Pt–Pd/PPy/ITO

**Fig. 2:**
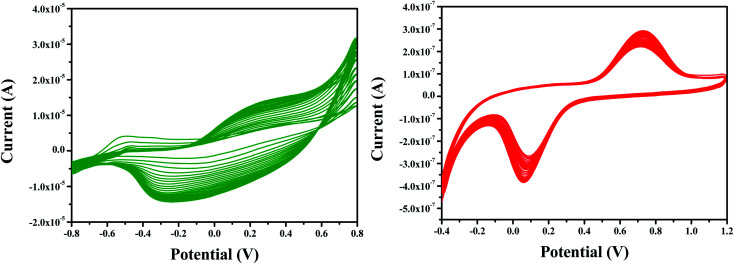
Cyclic voltammogram of electrodeposition of (a) PPy on ITO in pyrrole (0.01 M), SDS (0.01 M) and H_2_SO_4_ (0.01 M) at a scan rate of 50 mV s^−1^ for 25 cycles (b) Pt–Pd on PPy/ITO in H_2_PtCl_6_ (0.005 M) and PdCl_2_ (0.005 M) and H_2_SO_4_ (0.01 M) at a scan rate of 50 mV s^−1^ for 25 cycles.

Electropolymerization seems to be a promising technique for the polymerization of PPy on the electrode substrate as it provides a uniform coating. The cyclic voltammogram obtained for the electrochemical polymerization of pyrrole is shown in Fig. 2(a). Aqueous sulphuric acidic medium is used as an electrolyte containing 0.01 M pyrrole. The electropolymerization of PPy is performed in the potential range −0.8 V to 0.8 V at a scan rate of 50 mV s^−1^ for 25 cycles.

**Scheme 1:**
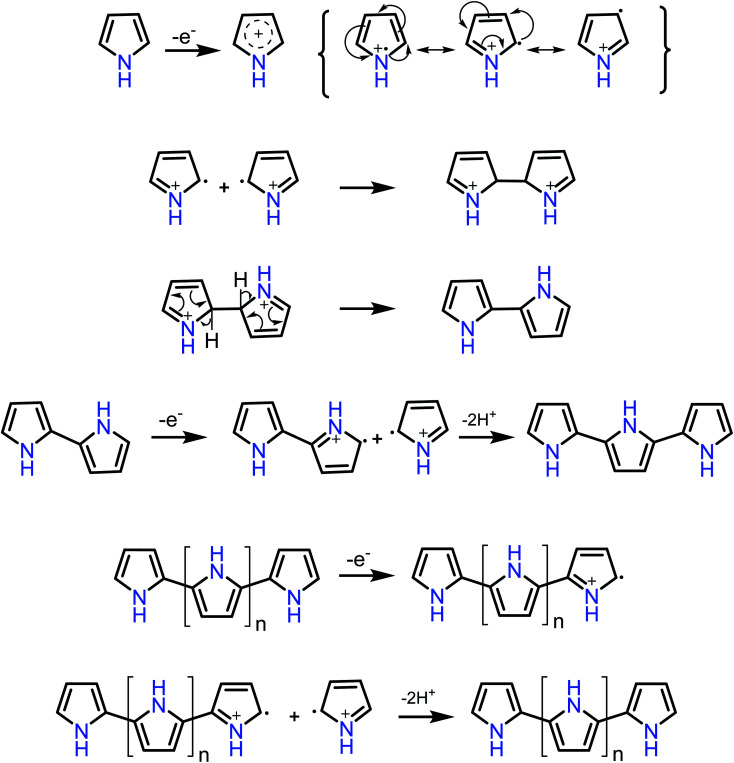
Probable mechanism of electro polymerization of pyrrole.

It is evident from the figure that oxidation of pyrrole occurs at a potential 0.2 V forming the first layer of polypyrrole. A very small current or nonexistence of current peak confirms that the further oxidation of polypyrrole is not happening. In the potential window applied, the polymer was found to be stable, and a reduction peak was observed at −0.4 V. The mechanism of polymerization is shown in Scheme 1.

With low solubility of monomers being a major drawback of synthesis of conducting polymers, an anionic surfactant like SDS is used to improve the solubility of pyrrole in aqueous acidic media. The high porous surface of polypyrrole provides active sites for the accumulation of bimetallic Pt–Pd nanoparticles. The dispersion of Pt–Pd nanoparticles on PPy/ITO electrode was performed by applying cyclic voltammetric technique between −0.4 to 1.2 V for 25 cycles *vs.* standard calomel electrode at 50 mV s^−1^ in an electrolyte encompassing 0.005 M of H_2_PtCl_6_ and PdCl_2_ each and 0.01 M of H_2_SO_4_. The cyclic voltammogram for the deposition of bimetallic Pt–Pd are shown in Fig. 2(b). Oxidation and reduction peaks were observed at 0.75 V and at 0.05 V respectively. Bimetallic Pt–Pd deposited electrodes were cleaned using double distilled water and kept in a desiccator overnight for drying. The modified electrode was used for anodic oxidation of 4-(hydroxymethyl) pyridine.

### 3.2. Voltammetric studies of potassium ferrocyanide/ferricyanide at Pt–Pd/PPy/ITO and PPy/ITO electrodes

**Fig. 3:**
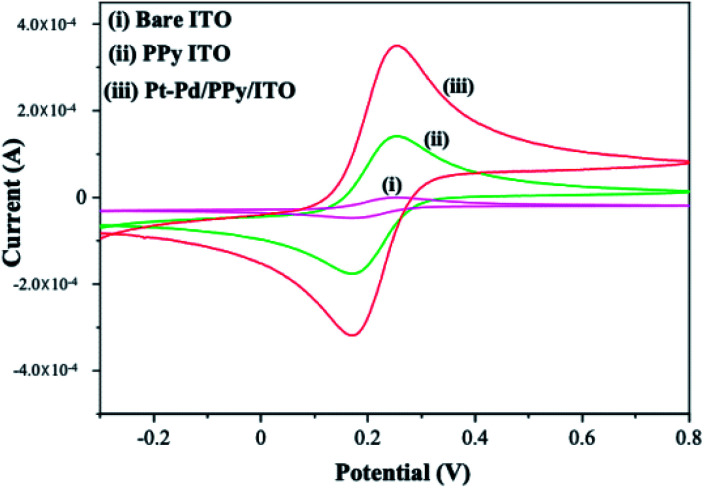
Cyclic voltammogram of 1 mM potassium ferrocyanide/ferricyanide at bare ITO (curve i) PPy/ITO (curve ii) Pt–Pd/PPy/ITO (curve iii) in 0.01 M H_2_SO_4,_ at a scan rate of 50 mV s^−1^.

Cyclic voltammograms of potassium ferri-ferrocyanide at Pt–Pd/PPy/ITO, PPy/ITO and bare ITO was studied at a scan rate of 50 mV s^−1^ (Fig. 3). Electrochemical active surface area of the electrode is calculated using the Randles–Sevcik equation using concentrations, respective slopes and diffusion coefficient.*i*_p_ = 2.69 × 10^5^*AD*^1/2^_0_*n*^3/2^*υ*^1/2^*C*

In the equation, *A* represents the electroactive surface area in cm^2^, *D*_0_ is the diffusion coefficient in cm^2^ s^−1^, *n* is the number of electrons participating in the reaction, *υ* is the scan rate in V s^−1^ and *C* is the concentration of potassium ferri-ferrocyanide in the bulk of the solution in mol cm^−3^. The electroactive surface area of electrode was estimated for bare ITO, PPy/ITO and Pt–Pd/PPy/ITO electrodes and were found to be 3.621 cm^2^, 5.183 cm^2^, 7.692 cm^2^ respectively.

The bare ITO electrode showed the least surface area. The highest surface area was shown by modified electrode Pt–Pd/PPy/ITO which is evident from the higher current density. Bare ITO showed least conductivity when compared to the modified electrodes. Incorporation of polymer species over bare ITO has helped to enhance the surface area as well as the conductivity of the surface. It also acts as a suitable host matrix for the incorporation of metal nanoparticles. Deposition of metal nanoparticles resulted in enhanced surface area which could be accounted for greater intensity in the redox peak (Fig. 3).

### 3.3. Electrochemical impedance studies

**Fig. 4:**
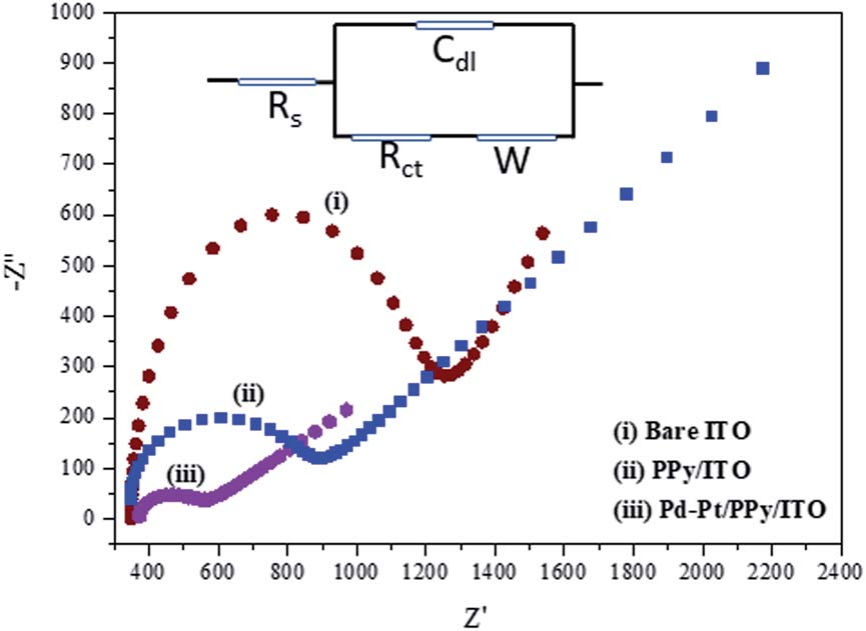
Nyquist plots of Pt–Pd/PPy/ITO, PPy/ITO and bare ITO in K_4_[Fe(CN)_6_] and 0.1 M KCl electrolyte.

Electrochemical impedance studies (EIS) give a better understanding regarding the charge transfer process occurring at the electrode–electrolyte interface. Fig. 4 reveals the Nyquist plots of Pt–Pd/PPy/ITO, PPy/ITO and bare ITO in 5 mM K_4_[Fe(CN)_6_] using 0.1 M KCl as electrolyte. Best fit Randel's equivalent electrical circuit model was developed for the EIS measurements data. The charge transfer resistance (*R*_ct_) will have a considerable change with the degree of adsorption of modifiers on the surface of the electrode, and it performs a significant role in controlling the electron transfer kinetics in a redox process of species K_4_[Fe(CN)_6_]/K_3_[Fe(CN)_6_]. The *R*_ct_ values on the surface of the electrode surface can be gauged by estimating the diameter of the semicircular region in the Nyquist plot. An increase in the slope of the Nyquist plot's linear segment provides evidence for the diffusion of K_4_[Fe(CN)_6_]/K_3_[Fe(CN)_6_] redox species towards the developed Pt–Pd/PPy/ITO electrode.

Surface modifications increases the double layer capacitance of the bare ITO which is clear from the EIS data. *R*_ct_ of bare ITO was found to be 1151 Ω. Incorporating a conducting polymer like PPy resulted in a considerable decrease in the *R*_ct_ value of 774.2 Ω, which accounts for the enhanced conductivity of PPy/ITO electrode than the bare ITO electrode. A further enhanced conductivity compared to PPy/ITO was shown by Pt–Pd/PPy/ITO having *R*_ct_ value of 438.9 Ω.

The *R*_ct_ values of electrodes were evaluated by fitting the EI spectra using the equivalent circuit, the order of *R*_ct_ is as follows Pt–Pd/PPy/ITO > PPy/ITO > bare ITO. This implies the significance of Pt and Pd nanoparticles in enabling the electron transfer at the electrode electrolyte interface. Impedance studies therefore prove that it is possible to deploy a modified ITO electrode as a better electrocatalyst for electrochemical oxidation of 4-(hydroxymethyl) pyridine.

### 3.4. SEM analysis

**Fig. 5:**
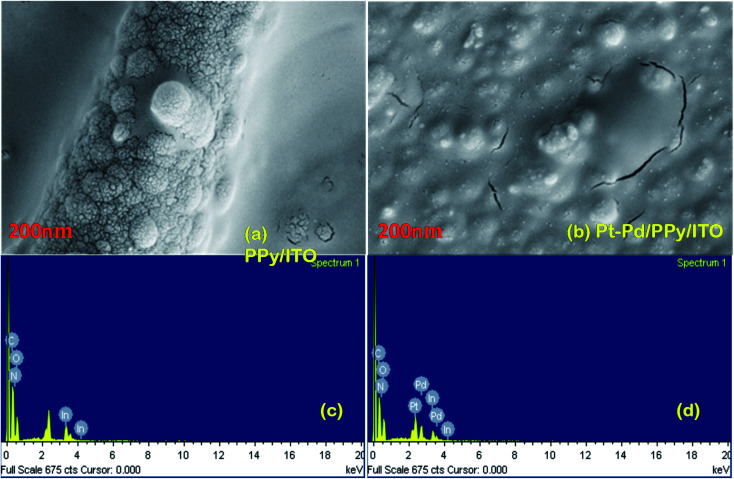
SEM images and EDS spectra of PPy/ITO (a and c respectively), Pt–Pd/PPy/ITO electrodes (b and d respectively).

SEM images of the PPy/ITO and Pt–Pd/PPy/ITO electrodes are shown in Fig. 5. PPy/ITO displayed a bulged vein like morphology which act as a uniform defect throughout the surface of electrode. These irregular distant bulged nano hemispheres act as the nucleation centre for the growth of the bimetallic Pt–Pd nuclei and further their growth. The distant nano hemispheres of PPy also facilitate the easy diffusion of electrolyte ions over the electrode surface. A tremor kind of morphology of bimetallic Pt–Pd was observed on the electrode surface. PPy veins were decorated by these uniform bimetallic Pt–Pd nanoparticles. The highly dispersed bimetallic nanoparticles and polymer chain are in proximity and thereby ensures high electrical conductivity through the polymer backbone to metal. The EDS spectra of PPy/ITO and Pt–Pd/PPy/ITO electrodes are depicted in the Fig. 5(c) and (d) respectively and confirmed the polymerization of PPy and deposition of Pt–Pd nanoparticles.

### 3.5. FTIR studies

**Fig. 6:**
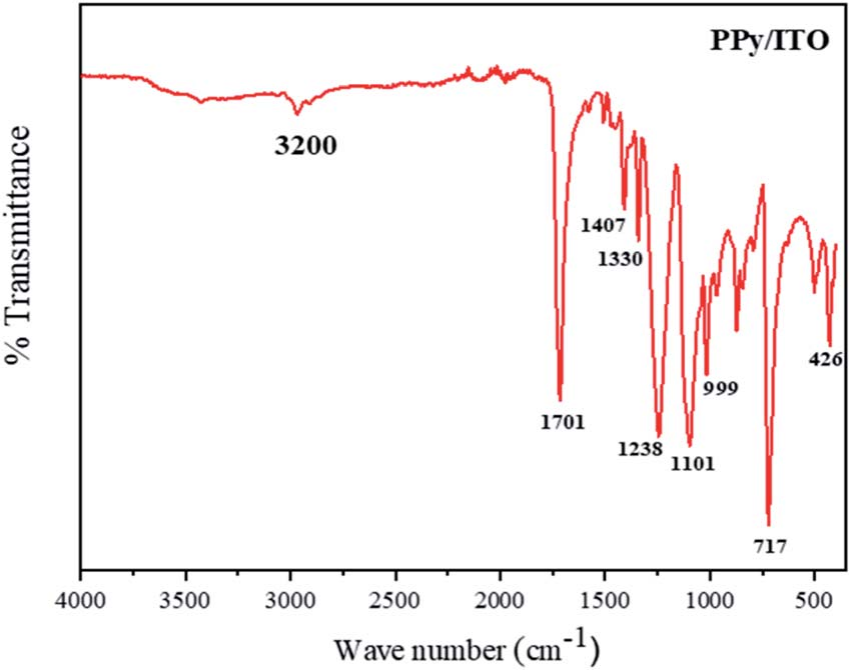
FTIR spectra of PPy/ITO electrode.

The IR peaks obtained for PPy/ITO, studied in a range between 400–4000 cm^−1^ as shown in Fig. 6. The major peaks obtained for the polymer are at 3200, 1701, 1407, 1330, 1238, 1101, 717, 426 cm^−1^. The peak at 3200 cm^−1^ can be attributed to the N–H stretching vibrations of the pyrrole ring. The characteristic peak at 1701 cm^−1^ corresponds to the C=N bond and the peaks at 1407 cm^−1^ and 1238 cm^−1^ can be ascribed to the C–N and C–H plane deformation bond respectively and the peak at 1101 cm^−1^ is the peak due to C–C stretching peak.

The peaks at 717 and 914 cm^−1^ are the peaks corresponding to the C–H wagging. The peaks observed were observed to be compliant with the existing literature.^9^

### 3.6. XRD analysis

**Fig. 7:**
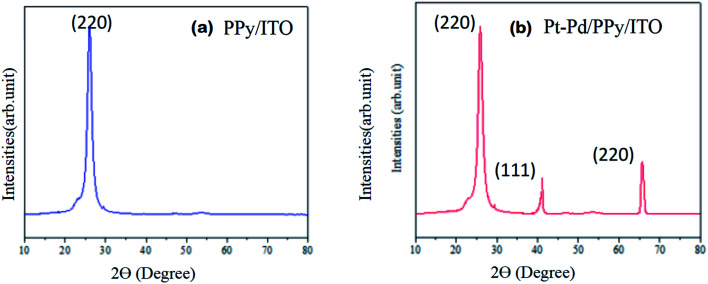
XRD patterns of modified electrodes (a) PPy/ITO and (b) Pt–Pd/PPy/ITO.

X-ray diffraction studies indicate amorphous behaviour of polypyrrole powder. As shown in Fig. 7 the peak shown at about 2*θ* = 26° is characteristic of amorphous polypyrrole and are due to scattering from polypyrrole chains at the interplanar space.^9,10^

The XRD pattern of bimetallic Pt–Pd/PPy/ITO has shown peaks at 2*θ* = 26.7, 40.1, 67.8 which are the characteristic peaks of Pt and Pd along with the peak of PPy. The peaks at 2*θ* = 26.7°, 40.1° and 67.8° can be attributed to (220), (111) and (220) planes respectively.

### 3.7. Surface study using optical profilometry technique

**Fig. 8:**
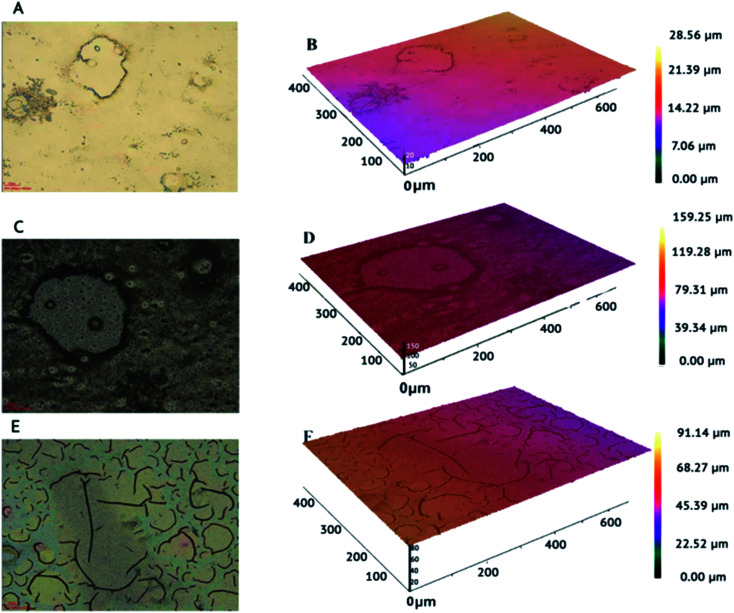
Optical profilometer images at magnification 20×: two dimensional (2-D) and three dimensional (3-D) images of bare ITO (a) and (b), PPy/ITO (c) and (d) and Pt–Pd/PPy/ITO (e) and (f) electrodes.

Surface topography of both bare ITO electrodes and modified were analyzed and studies using surface metrology technique optical profilometry. Zeta 3D images were analysed to study the dimensional and roughness details of the electrodes. Multidimensional images 2-D and 3-D of bare and modified electrodes obtained from optical profilometer at 20× magnification are represented in Fig. 8. Morphology of plane surface of bare ITO is given in Fig. 8(a) and (b). Root Mean Square (rms) roughness and standard deviation (SD) of bare ITO was calculated to be 0.4247 ± 0.0582 μm (Table 1). Fig. 8(c) and (d) depict 2-D image and 3-D profile of ITO coated with PPy. An enhanced surface roughness is observed from 0.4247 ± 0.0582 μm to 1.9319 ± 0.0408 μm (Table 1) after polymerization of PPy, which can be attributed to the materialization of a uniform rough film of PPy on the ITO electrode. Fig. 8(e) and (f) shows the formation of aggregates of nanoparticles of bimetallic Pt–Pd over the surface of PPy/ITO electrodes. Deposition of bimetallic nanoparticles even increased the surface area from 1.9319 ± 0.0408 μm to 4.5067 ± 0.2579 μm.

**Table 1:** Roughness of the bare ITO, PPy/ITO and Pt–Pd/PPy/ITO electrodes determined by optical profilometry

Electrode	rms roughness (μm)	SD (sigma)
Bare ITO	0.4247	0.0582
PPy/ITO	1.9319	0.0408
Pt–Pd/PPy/ITO	4.5067	0.2579

### 3.8. XPS analysis

**Fig. 9:**
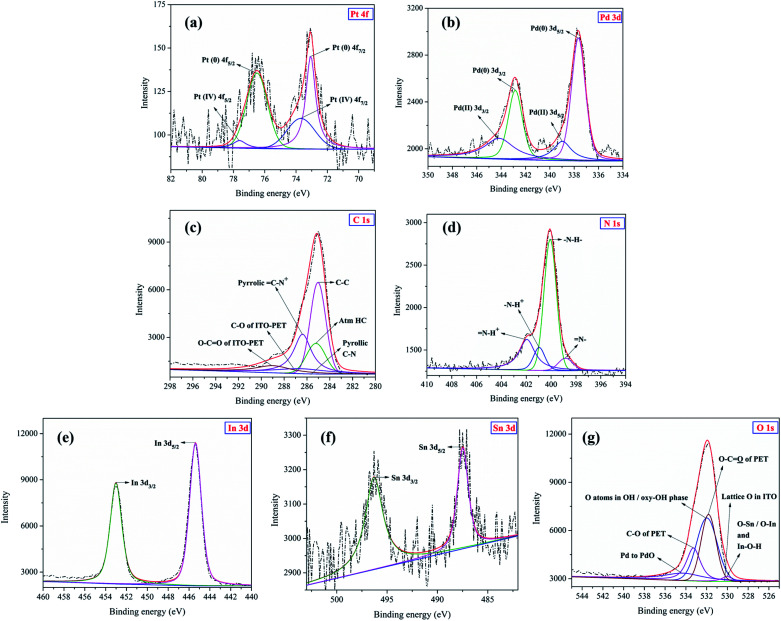
XPS spectra of (a) Pt 4f, (b) Pd 3d, (c) C 1s, (d) N 1s, (e) In 3d, (f) Sn 3d and (g) O 1s regions of Pt–Pd/PPy/ITO electrode used for electrochemical oxidation of 4-(hydroxymethyl) pyridine. Dotted lines in the figure show the original spectra, while solid lines depict the deconvoluted peaks. Solid red lines indicate the sum of deconvoluted peaks.

The XPS analysis spectra of Pt 4f, Pd 3d, C 1s, N 1s, In 3d, Sn 3d and O 1s regions of Pt–Pd/PPy/ITO electrode are shown in Fig. 9. These measurements were analysed to understand the valence state and surface elemental composition of Pt–Pd/PPy/ITO electrode. The survey spectrum revealed that Pt, Pd, C, N, In, Sn and O were present in the modified electrode (not shown). The core level XPS spectrum of Pt 4f region in Pt–Pd/PPy/ITO electrode is shown in Fig. 9(a). The typical peaks of Pt 4f valence states were observed at 73.1 eV and 76.4 eV assigned to Pt 4f_7/2_ and Pt 4f_5/2_ respectively.

A difference of about 3.3 eV for Pt 4f_5/2_ and Pt 4f_7/2_ peaks was seen. In addition, two less intense peaks present at 73.7 eV and 77.6 eV can be ascribed to Pt (iv) on electrode surface.^30^ In Fig. 9(b), the core level XPS spectrum of Pd 3d region displays doublets 3d_5/2_ and 3d_3/2_ at 337.6 eV (Pd (0))/338.9 eV (Pd (ii)) and 342.8 eV (Pd (0))/344.1 eV (Pd (ii)) respectively. A difference of about 5.2 eV for Pd 3d_3/2_ and Pd 3d_5/2_ at 0 and (ii) oxidation states was observed which is typical of Pd. The deconvoluted peaks belonging to Pd in 0 and (ii) oxidation states were asymmetric and (ii) oxidation state was as a result of surface oxidation (Pd to PdO formation).^31^ The XPS spectrum of C 1s was depicted in Fig. 9(c). The peak observed at 285.0 eV belonged to C–C bonds present in PPy as well as ITO-PET substrate. The presence of a thin layer of hydrocarbons in nano dimensions belonging to ITO-PET on exposure to air was corroborated from peak obtained at 285.2 eV.^32^ The peaks observed at 285.7 eV and 286.3 eV indicate the presence of pyrrolic CN bond and oxidized =C–N^+^ moiety.^33^ Two peaks assigned to different chemical environments of carbon in ITO-PET, C–O and O–C=O were seen at 286.5 eV and 289.0 eV respectively.^34^ Two types of N 1s peaks belonging to PPy were observed at 398.8 eV (sp^2^) and 400.0 eV (sp^3^) in the N 1s XPS spectrum Fig. 9(d). The peaks at 400.0 eV, 400.9 eV and 401.9 eV belonged to –N–H– (neutral), oxidized –N–H^+^ and =N–H^+^ moieties of PPy respectively.^33^

The XPS spectrum of In 3d displayed peaks at 445.3 eV and 453.0 eV which can be ascribed to In 3d_5/2_ and In 3d_3/2_ respectively present on ITO-PET substrate Fig. 9(e).^35^ The XPS spectrum of Sn 3d indicated Sn 3d_5/2_ and Sn 3d_3/2_ peaks at binding energy values 487.4 eV and 496.3 eV respectively Fig. 9(f).^35^ In Fig. 9(g), the XPS core level spectrum of O 1s displays two peaks at 530.1 eV and 530.3 eV corresponding to oxygen atoms in hydroxide or oxyhydroxide phase and lattice oxygen present in ITO-PET respectively.^36^ O bonded to In and/or Sn and In–O–H was seen at binding energy value 532.0 eV.^32^ Additional peaks at 531.8 eV and 533.2 eV were seen corroborating O–C=O and C–O of ITO-PET respectively.^37^ Also, the peak at 534.2 eV binding energy supports partial surface oxidation of Pd to PdO.^38^

**Fig. 10:**
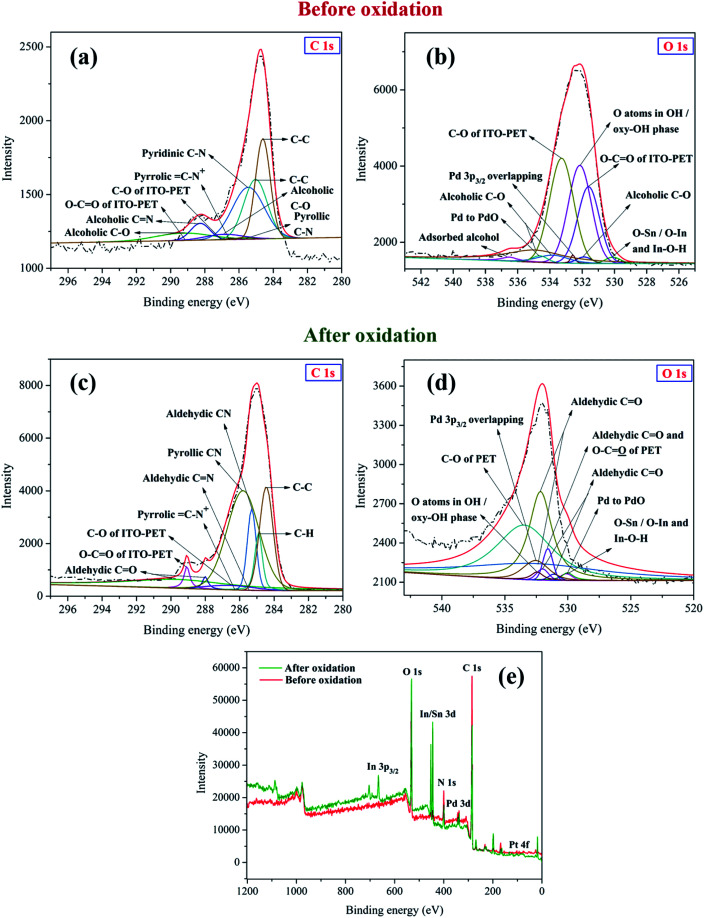
The XPS spectra of (a) C 1s and (b) O 1s regions of Pt–Pd/PPy/ITO electrode before oxidation of 4-(hydroxymethyl) pyridine. The XPS spectra of (c) C 1s and (d) O 1s regions of Pt–Pd/PPy/ITO electrode after oxidation of 4-(hydroxymethyl) pyridine. (e) The XPS wide scan spectra of Pt–Pd/PPy/ITO electrode used before and after oxidation of 4-(hydroxymethyl) pyridine. Dotted lines indicate original spectra, while solid lines are indicative of the deconvoluted peaks. Red solid lines depict the sum of deconvoluted peaks.

The XPS measurements were performed for Pt–Pd/PPy/ITO electrode used before and after oxidation of 4-(hydroxymethyl) pyridine Fig. 10. The XPS spectra of C 1s and O 1s regions of the modified electrode prior to oxidation are depicted in Fig. 10(a) and (b) respectively. The core level C 1s XPS spectrum displayed a peak at 284.6 eV belonging to C–C or C=C present in PPy and 4-(hydroxymethyl) pyridine in Fig. 10(a).^33–38^ The peaks observed at 285.0 eV and 285.2 eV can be assigned to C–C of ITO-PET substrate.^34^ C–N and C=N moieties present in 4-(hydroxymethyl) pyridine adsorbed on Pt–Pd/PPy/ITO used before oxidation were corroborated from peaks at 285.4 eV and 288.3 eV respectively.^39^ The peaks around 285.6 eV and 286.4 eV indicate the presence of pyrrolic CN bond and oxidized =C–N^+^.^33^

The peaks corresponding to C–O and O–C=O of ITO-PET substrate were observed at 286.5 eV and 289.1 eV respectively.^34^ Most importantly, two peaks at 286.8 eV and 289.2 eV corroborate the presence of C–O bond in hydroxyl group of 4-(hydroxymethyl) pyridine.^39^ The XPS signal for O 1s seen in Fig. 10(b) indicate peak at 530.0 eV that can be assigned to O bonded to In and/or Sn and In–O–H.^32^ The presence of alcoholic C–O bond in 4-(hydroxymethyl) pyridine can be inferred from peaks obtained at 531.8 eV, 533.8 eV and 534.9 eV.^40^ The molecular alcohol was present as indicated by a peak at binding energy 536.5 eV.^41^ The peaks at 531.6 eV and 533.2 eV reveals two types of chemical environments of oxygen, O–C=O and C–O of ITO-PET respectively.^37^ The peak at 532.1 eV correspond to oxygen atoms in hydroxide or oxyhydroxide phase present in ITO-PET substrate.^36^ Additional peak at 532.6 eV was seen as a result of Pd 3p_3/2_ overlapping.^42^ Pd to PdO surface oxidation can be inferred from peak at 534.6 eV.^38^

Fig. 10(c) and (d) respectively show the XPS spectra of C 1s and O 1s regions of the modified electrode after oxidation. The peaks observed at 284.4 eV and 284.9 eV are related to C–C/C=C and C–H bonds respectively belonging to PPy and 4-formyl pyridine.^33–38^ The peaks related to C–C, C–O and O–C=O of ITO-PET substrate can be attributed to binding energy values 285.0 eV, 286.6 eV and 289.0 eV respectively.^34^ The peaks at 285.3 eV and 285.5 eV refer to CN bond present in 4-formyl pyridine.^39^ Pyrrolic CN bond and oxidized =C–N^+^ are confirmed from peaks at 285.8 eV and 286.3 eV respectively.^33^ The peaks obtained at 288.0 eV and 289.6 eV can be ascribed to aldehydic C=O groups of 4-formyl pyridine.^43^Fig. 10(d) represents the XPS core level spectrum of O 1s of Pt–Pd/PPy/ITO electrode used after oxidation of 4-(hydroxymethyl) pyridine. At 530.1 eV, O bonded to In and/or Sn was observed,^32^ whereas the peak at 530.0 eV indicates partial surface oxidation of Pd to form PdO.^44^ The peaks at 531.1 eV, 532.0 eV, 532.1 eV and 532.7 eV can be attributed to the presence of aldehydic C=O moiety present in 4-formyl pyridine.^43^ The peak at 531.6 eV can be ascribed to both aldehydic C=O group and O–C=O group present in 4-formyl pyridine and ITO-PET substrate respectively.^37–43^ The presence of oxygen atoms in hydroxide or oxyhydroxide phase in ITO-PET substrate can be inferred from peak at 532.4 eV.^36^ C–O of ITO-PET was corroborated by XPS signal obtained at 533.4 eV.^37^ Pd 3p_3/2_ overlap was seen at 532.5 eV.^42^ One prominent observation was the absence of molecular alcohol at binding energy 536.5 eV in the O 1s spectra for the modified electrode used after oxidation. The XPS wide scan spectral analysis of Pt–Pd/PPy/ITO electrodes used before and after oxidation of 4-(hydroxymethyl) pyridine is depicted in Fig. 10(e), which indicates slight changes in the binding energy values with respect to C 1s and O 1s regions. Therefore, the above analysis results evidently favour electrochemical oxidation of 4-(hydroxymethyl) pyridine to 4-formyl pyridine.

### 3.9. Electrocatalytic oxidation of 4-(hydroxymethyl) pyridine

**Fig. 11:**
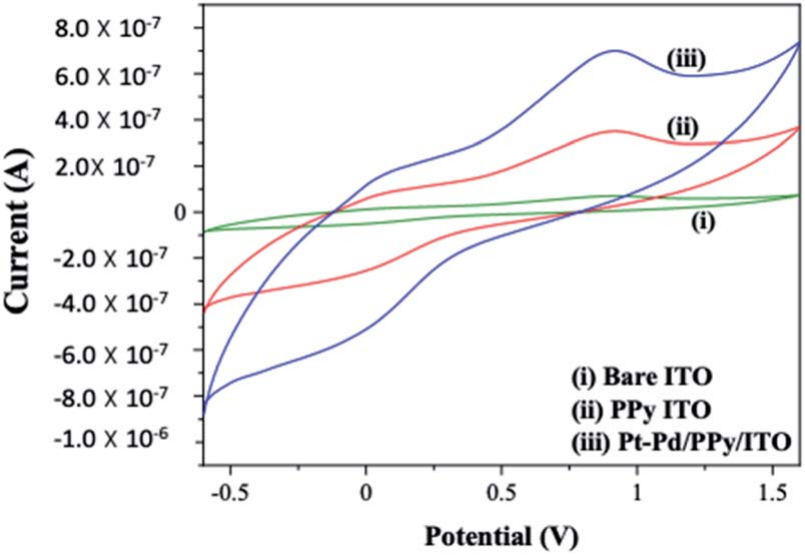
Electrocatalytic oxidation of 4-(hydroxymethyl) pyridine using (i) bare ITO (ii) PPy/ITO and (iii) Pt–Pd/PPy/ITO in aqueous H_2_SO_4_ medium (0.01 M).

**Scheme 2:**
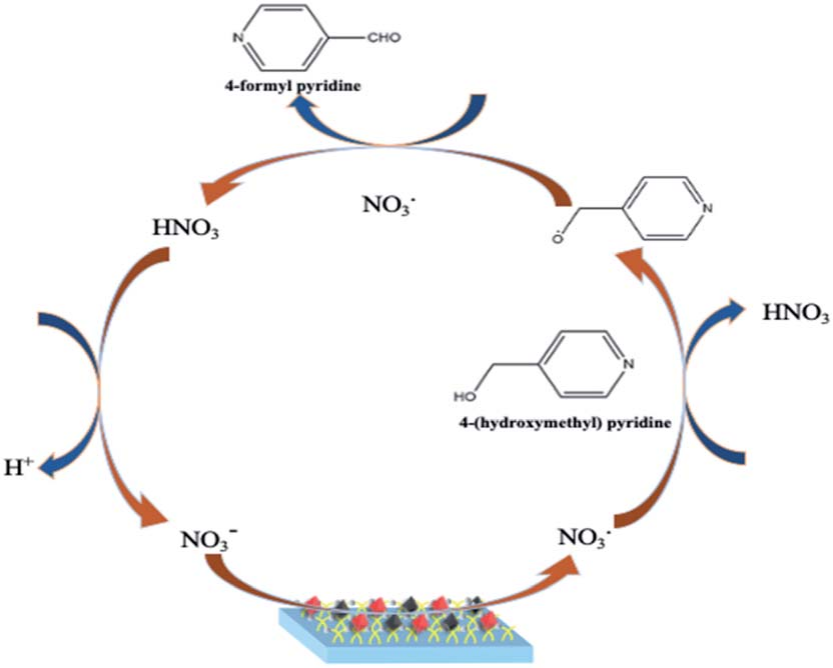
Mechanism of electrocatalytic oxidation of 4-(hydroxymethyl) pyridine.

Cyclic voltammetric experiments were conducted to assess the electrocatalytic oxidation of 4-(hydroxymethyl) pyridine using bare ITO, PPy/ITO and Pt–Pd/PPy/ITO electrodes and represented in Fig. 11. Sodium nitrate (10 mM) is used as a mediator for the reaction. The experiments were conducted at a scan rate of 50 mV s^−1^ kin an aqueous acidic medium. An anodic peak is obtained at 0.903 V with a peak current 6.7801 × 10^−8^ A cm^−2^ for the electrooxidation of 4-(hydroxymethyl) pyridine at bare ITO electrode. An increase in anodic peak current (7.4148 × 10^−7^ A cm^−2^) was observed for Pt–Pd/PPy/ITO electrode towards alcohol oxidation. Electrochemical activity of the modified electrodes is improved due to enhanced surface area of the modified electrodes. Alcohol molecules are largely attracted to the electroactive surface of the modified electrode because of Pt–Pd bimetallic nanoparticles generating high energy active sites. The plausible reaction mechanism is given in Scheme 2. Nitrate ions in sodium nitrate gets oxidized at the anode forming nitrate radical. The reaction is then followed by the abstraction of hydroxyl proton from the 4-(hydroxymethyl) pyridine leading to the formation of 4-(hydroxymethyl) pyridine radical. Alcohol radical is again attacked by the nitrate radical leading to the abstraction of another hydrogen radical results in the formation of the corresponding aldehyde. The reaction is carried out in an aqueous acidic medium, the acid plays no role in the reaction mechanism. It acts as an electrolyte and provides better conductivity.

**Fig. 12:**
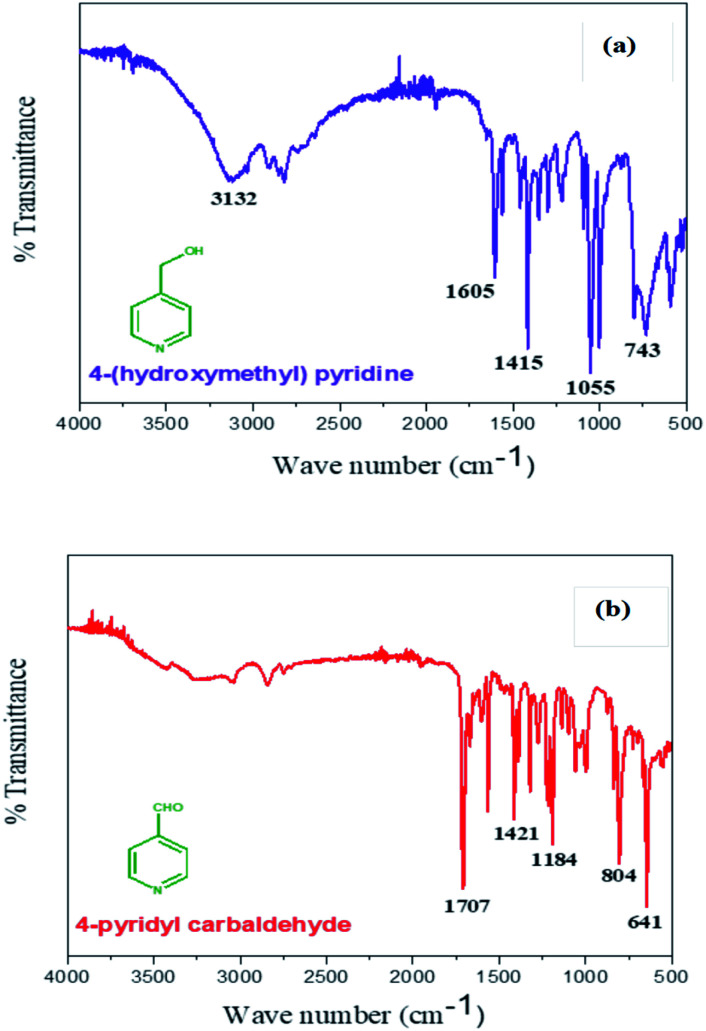
FTIR spectra of (a) 4-(hydroxymethyl) pyridine and (b) 4-formyl pyridine.

GC-MS was employed to confirm the completion of electrochemical oxidation reaction. The product obtained after the prolonged electrolysis of 4-(hydroxymethyl) pyridine (10 mM) is analyzed by GC-MS using a Shimadzu instrument (Model GCMS-QP2010 SE). The purity of the product is estimated as 74% and the study shows that the electrode can be effectively used to convert 4-(hydroxymethyl) pyridine into 4-formyl pyridine. The purified product was investigated by FTIR spectroscopy and Fig. 12(a) depicts the IR data of reactant 4-(hydroxymethyl) pyridine and Fig. 12(b) shows the IR data of product 4-formyl pyridine. The peak at 3132 cm^−1^ for reactant (Fig. 12(a)) corresponds to the hydroxyl group of 4-(hydroxymethyl) pyridine which is absent in the IR data of product (Fig. 12(b)). Additional peak at 1707 cm^−1^ attributes the carbonyl group and this confirms the formation of 4-formyl pyridine.

### 3.10. Effect of scan rate

**Fig. 13:**
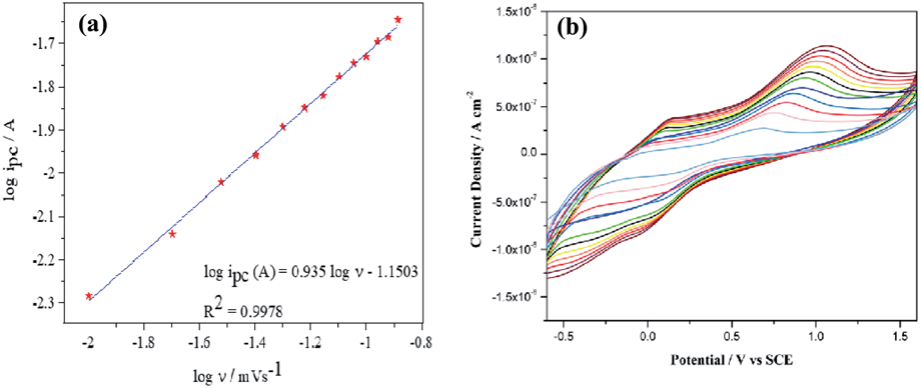
(a) Cyclic voltammogram of oxidation of 4-(hydroxymethyl) pyridine at Pt–Pd/PPy/ITO electrodes with scan rates varying from 10 mV s^−1^–130 mV s^−1^ (b) plot of logarithm of anodic peak currents *vs.* logarithm of scan rates.

The effect of scan rate was studied for the 4-(hydroxymethyl) pyridine oxidation in an aqueous acidic condition in presence of sodium nitrate using modified Pt–Pd/PPy/ITO electrodes. Cyclic voltammetric studies were done by 4-(hydroxymethyl) pyridine (10 mM) for different scan rates ranging from 10 mV s^−1^ to 130 mV s^−1^ are shown in Fig. 13(a). The logarithm of peak current was charted against that of scan rates and the linear plot obtained (Fig. 13(b)) indicates adsorption controlled electrochemical process.

### 3.11 Optimisation of number of cycles

**Fig. 14:**
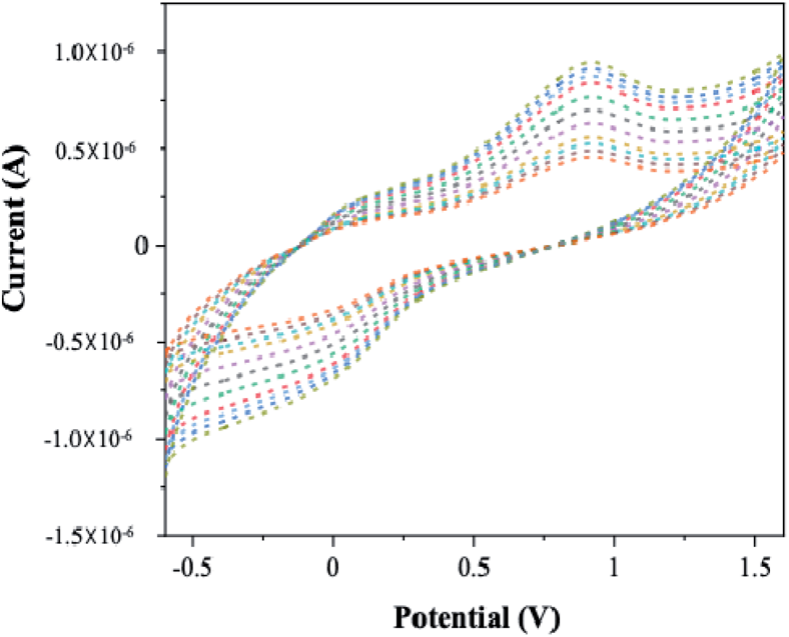
Effect of number of cycles for the electrochemical oxidation of 4-(hydroxymethyl) pyridine using Pt–Pd/PPy/ITO electrodes.

Electrochemical oxidation of 4-(hydroxymethyl) pyridine (10 mM) in sodium nitrate medium using Pt–Pd/PPy/ITO electrode was observed for a set of cycles such as 3, 5, 8, 10, 12, 15, 18, 20, 22, 24 and 25 needed for the deposition of bimetallic Pt–Pd on PPy coated ITO electrode represented in Fig. 14. A linear increase in the anodic peak current was detected for the oxidation of the alcohol. The potential was found to be stable and shifted towards the positive values up to 25 cycles, after which the potential values remain unchanged. Therefore, 25 cycles were considered as the optimal number for the bimetallic Pt–Pd deposition on PPy/ITO electrodes for 4-(hydroxymethyl) pyridine oxidation. The increase in the peak potentials towards positive values are attributed to the surface adsorption at the electrode interface.

### 3.12 Stability and repeatability

**Fig. 15:**
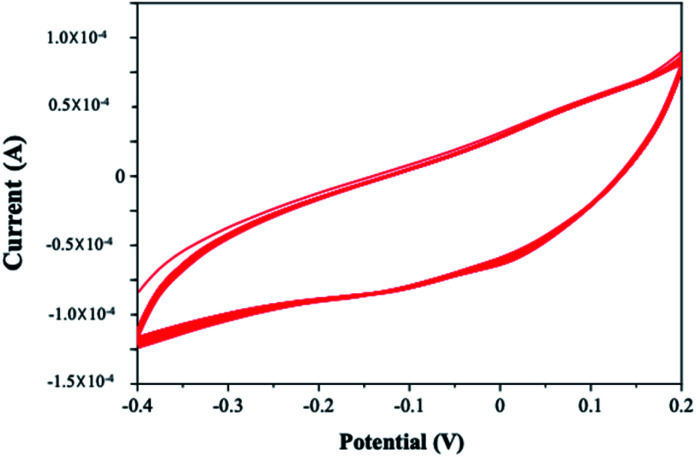
Cyclic voltammogram of 50 continuous cycles of Pt–Pd/PPy/ITO in the absence of 4-(hydroxymethyl) pyridine in 0.01 M H_2_SO_4_ and distilled water.

To ensure the performance of the Pt–Pd/PPy/ITO electrode for future application as an effective electrode for electrocatalytic oxidation, the reproducibility of the electrode is tested by carrying out repeated electrocatalytic oxidation of 4-(hydroxymethyl) pyridine (10 mM) five times in a month with a time interval of 7 days. Six electrodes were prepared and tested in presence of 4-(hydroxymethyl) pyridine. Electrodes showed better stability for a long run in nitrate mediated electrocatalytic oxidation of 4-(hydroxymethyl) pyridine. A similar study is performed in the absence of 4-(hydroxymethyl) pyridine, with H_2_SO_4_ in aqueous media using Pt–Pd/PPy/ITO electrodes to confirm the stability of modified electrode which is shown in Fig. 15.

## 4 Conclusions

This study explored the catalytic efficiency of the immobilised polypyrrole conducting with bimetallic Pt–Pd nanoparticles. The electrode displayed exceptional electrocatalytic activity and good stability for the complete oxidation of 4-(hydroxymethyl) pyridine to 4-formyl pyridine. Furthermore, the modified electrode showed high reproducibility and required mild reaction conditions, avoiding the use of chemical oxidants. Radical initiated reaction *via* electrochemical methods are a reliable and efficient method for the generation of radicals. The conclusions section should come in this section at the end of the article, before the acknowledgements.

## Conflicts of interest

The authors confirm they do not have any conflicts of interest regarding this manuscript.

## Supplementary Material
